# Determination of Tissue Potassium and Sodium Concentrations in Dystrophic Skeletal Muscle Tissue Using Combined Potassium (^39^K) and Sodium (^23^Na) MRI at 7 T

**DOI:** 10.1002/nbm.70009

**Published:** 2025-02-17

**Authors:** Lena V. Gast, Teresa Gerhalter, Matthias Türk, Alper Sapli, Claudius S. Mathy, Rafael Heiss, Pierre‐Yves Baudin, Benjamin Marty, Michael Uder, Armin M. Nagel

**Affiliations:** ^1^ Institute of Radiology, University Hospital Erlangen Friedrich‐Alexander‐Universität Erlangen‐Nürnberg (FAU) Erlangen Germany; ^2^ Department of Neurology, University Hospital Erlangen Friedrich‐Alexander‐Universität Erlangen‐Nürnberg (FAU) Erlangen Germany; ^3^ Centre for Rare Diseases Erlangen (ZSEER), University Hospital Erlangen Friedrich‐Alexander‐Universität Erlangen‐Nürnberg (FAU) Erlangen Germany; ^4^ NMR Laboratory, Neuromuscular Investigation Center Institute of Myology Paris France; ^5^ Division of Medical Physics in Radiology German Cancer Research Center (DKFZ) Heidelberg Germany

**Keywords:** 7 T, facio‐scapulo‐humeral muscular dystrophy, potassium MRI, skeletal muscle, sodium MRI, ultrahigh field strength

## Abstract

Combined ^23^Na/^39^K MRI at 7 T can highlight ion disturbances in skeletal muscle tissue. In this work, we investigated if the apparent tissue potassium concentration (aTPC) can be determined in fatty replaced muscles of patients with facio‐scapulo‐humeral muscular dystrophy (FSHD) and if it can provide additional information to the fat replacement and the apparent tissue sodium concentration (aTSC). The lower leg of 14 patients (six females, eight males; mean age 47.7 ± 14.0 years) with genetically confirmed FSHD and 11 healthy controls (four females, seven males; mean age 47.0 ± 14.0 years) was examined at a 7‐T MR system using a dual‐tuned ^23^Na/^39^K birdcage RF coil. In addition, qualitative and quantitative ^1^H MR measurements were performed at 7 T to assess the fat replacement and water accumulation. The aTPC and aTSC were determined in seven different muscle regions based on five external references phantoms and corrected for partial volume effects, relaxation biases, and reduced ion concentrations in fat. Results are expressed as median (interquartile range). The measured aTPC was strongly reduced in fat‐replaced muscles and was close to zero in totally fat replaced muscles (aTPC = 4.3 mM [2.7 mM] for FF > 80%). After correction of aTPC values for reduced potassium concentration in fat, aTPC_fc_ values of patients in muscles with low or moderate fat fraction (FF < 30%) were similar to values of healthy subjects (patients: aTPC_fc_ = 85.6 mM [21.7 mM]; controls: aTPC_fc_ = 83.2 mM [22.3 mM]). However, muscles with FF > 30% showed reduced aTPC_fc_ and increased aTSC_fc_ compared with healthy controls (aTPC_fc_ = 28.9 mM [46.2 mM], aTSC_fc_ = 42.3 mM [17.6 mM]; controls: aTSC_fc_ = 15.0 mM [4.6 mM], aTPC_fc_ = 83.2 mM [22.3 mM]). No correlations were observed between the aTPC_fc_ and aTSC_fc,_ or between aTPC_fc_ and water T_2_. We showed that a determination of the aTPC in dystrophic skeletal muscles is feasible using ^39^K MRI at 7 T. Measured changes in aTPC_fc_ were greater than sole fat replacement and might therefore be used as an additional quantitative measure for dystrophic muscle tissue.

## Graphical abstract

The apparent tissue potassium concentration (aTPC) measured by ^39^K MRI in fat‐replaced muscles of patients with neuromuscular disorders is impacted by the low concentration of potassium in fat. To assess the aTPC in remaining muscle tissue, we correct this impact using the intramuscular fat fraction (FF) measured by ^1^H MRI.

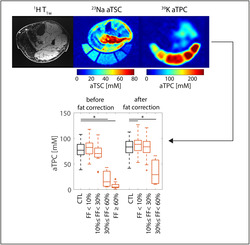

**Abbreviations**
aTSC: apparent tissue sodium concentration
aTPC: apparent tissue potassium concentration
CTL: healthy control
DA‐3D‐RAD‐C: three‐dimensional density‐adapted radial sequence with cubic field‐of‐view
EDL: extensor digitorum longus muscle
FF: fat fraction
FC: fat‐corrected
FL3D: three‐dimensional fast low angle shot sequence
FS: fat‐suppressed
FSHD: facio‐scapulo‐humeral muscular dystrophy
GM: gastrocnemius muscle, medial head
GL: gastrocnemius muscle, lateral head
MESE: multi‐echo spin‐echo
PVC: partial volume correction
RF: radiofrequency
PER: peroneus muscle
SOL: soleus muscle
TA: tibialis anterior muscle
TSE: turbo spin echo
T_1w_
: *T*_1_‐weighted
T_2w_
: *T*_2_‐weighted
WE: water‐excitation
^23^Na MRI: sodium magnetic resonance imaging
^39^K MRI: potassium magnetic resonance imaging

## 1 Introduction

Sodium (Na^+^) and potassium (K^+^) ions play an essential role in the excitability and homeostasis of muscle cells due to their concentration gradients between the intracellular and extracellular spaces. Various diseases, including muscular dystrophies, have been reported to alter the ion distributions [1, 2]. Sodium (^23^Na) MRI has already been successfully applied to noninvasively investigate changes in the muscular sodium concentrations of muscular dystrophy patients, for example, in Duchenne muscular dystrophy [3, 4] or facioscapulohumeral muscular dystrophy (FSHD) [5]. Typically, ^23^Na MRI measures the apparent tissue sodium concentration (aTSC), which represents the average of intracellular and extracellular sodium contents, weighted by the respective volume fractions of the intracellular and extracellular spaces. In addition, specific techniques such as inversion recovery ^23^Na MRI have been developed which are more sensitive toward changes in the intracellular sodium content [6]. Particularly, in patients with Duchenne muscular dystrophy, an enhanced aTSC has been suggested as an early biomarker for the disease activity [4].

Similarly, potassium (^39^K) MRI can be used to noninvasively measure the apparent tissue potassium concentration (aTPC) in skeletal muscle tissue [7]. As skeletal muscle is the most important storage of potassium within the human body containing approximately 75% of the total potassium content [8], a noninvasive K^+^ concentration determination in human skeletal muscle tissue is highly desirable. In particular, the combination of ^23^Na and ^39^K MRI could provide interesting insights into the ion homeostasis of pathologic skeletal muscles due to the inverse concentration distributions of these ions between the intracellular and extracellular spaces.

However, the MR sensitivity of ^39^K nuclei is strongly reduced compared with ^23^Na and conventional proton (^1^H) MRI, and the resulting very low signal‐to‐noise ratio of ^39^K MRI requires the use of ultra‐high‐field MR systems (≥ 7 T). Moreover, ^39^K nuclei in muscle tissue possess very short transverse relaxation times (T_2short_* = 1.2, T_2long_* = 8.1 ms), and their signal decay is influenced by residual quadrupolar interactions [7, 9], making the detection as well as quantification of the ^39^K MR signal challenging. Recent technical advances have made it possible to quantify aTPC values in skeletal muscle tissue with high repeatability [9]. Still, ^39^K MRI is rarely used in humans and its application has been mostly limited to healthy muscle and brain tissue so far [7, 9, 10, 11, 12, 13, 14]. In contrast, ^23^Na MRI has already been applied in a large variety of different clinical studies including various muscular pathologies [15, 16]. Na^+^ and K^+^ concentrations in fatty tissue are reduced compared with muscle tissue, with an almost negligible K^+^ concentration in fat [17]. To assess the aTSC values in remaining muscle tissue of partially fat replaced muscles, a correction using the muscular fat fraction (FF) has been proposed [4].

In this work, we applied quantitative ^39^K MRI together with ^23^Na MRI and ^1^H MRI at 7 T to examine the lower leg muscle tissue of FSHD patients as a model for dystrophic skeletal muscle tissue. The aberrant expression of the transcription factor DUX4 in FSHD patients leads to muscle damage, with asymmetric muscle involvement, especially in the initial phase of disease progression. Patients show progressive muscle wasting described by fat replacement initiated by inflammation and edema [18]. Also, metabolic and ion disturbances have been described [5, 19].

The aim of this work was to investigate the feasibility of aTPC determination in partly fat‐replaced dystrophic skeletal muscle tissue and its potential benefit compared with other commonly applied quantitative MRI measures such as the aTSC, FF, and water T_2_ to assess dystrophic muscle tissue.

## 2 Methods

### 2.1 Study Population

Fourteen patients with genetically proven FSHD1 (six female subjects, eight male subjects; mean age 47.7 ± 14.0 years, range 25–67 years) were enrolled in this prospective MRI study. In addition, 11 healthy controls (four female subjects, seven male subjects; mean age 45.4 ± 13.5 years, range 25–64 years) were recruited. Exclusion criteria were contraindications for MRI (e.g., claustrophobia or metallic implants) and an age younger than 18. The study was approved by the local ethical review board (No: 342_16 B, Erlangen, Germany), and written informed consent was obtained from all subjects. Examinations were performed between April 2019 and March 2021.

### 2.2 Muscle Strength Grading

In all patients, a neurologist scored manually the strength using the Medical Research Council grading system [20] ranging from 0 (*no muscle contraction*) to 5 (*maximal muscle strength*) for the foot dorsal and plantar flexors. The neurologist also collected clinical data (age at disease onset, total disease duration, duration of muscle weakness of lower legs, mutation, and medication) by a personal interview and from available medical records.

### 2.3 MRI Examinations

All MRI examinations were performed on a whole‐body 7 T MRI system (Magnetom Terra, Siemens Healthineers AG, Erlangen, Germany). For ^23^Na/^39^K MRI, a double‐resonant birdcage transmit/receive radiofrequency (RF) coil (Rapid Biomedical GmbH, Rimpar, Germany) was used. As the measurement setup for ^23^Na/^39^K MRI did not contain a ^1^H channel, *B*_0_ shimming was performed based on the ^23^Na MR signal [21]. In addition, ^1^H MRI data of the subjects were acquired using a one‐channel transmit and 28‐channel receive knee RF coil (Quality Electrodynamics, Mayfield Village, OH, USA).

In patients, MR datasets were acquired from the less affected leg (compare Table 1). In healthy subjects and patients with no difference in leg muscle strength, the right leg was scanned. Subjects were examined in feet first supine position. For ^23^Na/^39^K MRI, the leg was positioned directly on a reference phantom holder consisting of five compartments that were filled with different combinations of NaCl and K_2_HPO_4_ solution ([Na^+^]/[K^+^] = 10/240, 20/210, 25/180, 30/150, 40/120 mM). The biggest circumference of the calf was placed within the isocenter of the ^23^Na/^39^K RF coil and marked to ensure the same positioning for the ^1^H MRI examinations. Between the acquisition of the ^23^Na/^39^K and ^1^H MRI datasets, a change of RF coil and repositioning of the subjects was necessary.

**TABLE 1:** Patient demographics and clinical data of examined FSHD patients.

Patient	Sex	Age	Disease duration (years)	Stronger leg (left/right)	Dorsal extension	Plantar flexion
1	w	60	>50	r	1	4
2	w	70	42	r	0–1	4+
3	w	35	21	r	5	5
4	m	58	18	l	5	5
5	m	49	39	l	2	5
6	w	33	>25	r	5	5
7	m	67	>50	r	2	3–4
8	w	31	21–25	r	5	5
9	w	25	15–19	l	5	5
10	m	61	2	l	5	5
11	m	33	8	r	5	5
12	m	46	30	r	4+	4+
13	m	45	5–30	l	3	4+
14	m	55	25	l	2	5
Mean		48 ± 14		Median	4.5	5

^23^Na and ^39^K MR images were acquired using a three‐dimensional density‐adapted radial sequence with cubic field‐of‐view (DA‐3D‐RAD‐C) (see Table 2 for measurement parameters) [22, 23]. This readout scheme enables the acquisition of anisotropic voxels at ultra‐short echo times, which is beneficial for the examination of ^23^Na/^39^K in skeletal muscles. The ^1^H MRI protocol consisted of an ultrahigh‐resolution T_1_‐weighted (T_1w_) water‐excitation (WE) 3D FLASH (FL3D) sequence, a T_2_‐weighted (T_2w_) fat‐suppressed (FS) turbo spin‐echo (TSE) sequence, and a multi‐echo spin‐echo (MESE) sequence for mapping of water T_2_ relaxation times and determination of the intramuscular FF.

**TABLE 2:** Acquisition parameters for quantitative ^**23**^Na and ^**39**^K MRI acquired using a 3D density‐adapted radial readout scheme with cubic field‐of‐view (DA‐3D‐RAD‐C) as well as ^**1**^H MRI performed using T_1w_ FL3D with WE, T_2w_ TSE with FS, and MESE sequences at 7 T.

	^23^Na DA‐3D‐RAD‐C	^39^K DA‐3D‐RAD‐C	^1^H T_1w_ FL3D	^1^H T_2w_ TSE	^1^H MESE
TR (ms)	120	40	11	9990	3000
TE (ms)	0.3	0.4	4.58	54	9.5–304 (32 echoes)
Flip angle (°)	90	90	7	150	90–180
Nominal spatial resolution (mm^3^)	2.5 × 2.5 × 15	8 × 8 × 32	0.1 × 0.1x1	0.23 × 0.23 × 2	1 × 1 × 10
Number of projections	5384	2960	—	—	—
Number of slices	—	—	224	42	5
BW (Hz/Px)	—	—	270	202	449
Readout duration (ms)	10	5	—	—	—
*T*_Acq_ (min:s)	10:46	09:52	11:32	7:11	03:41

### 2.4 Data Evaluation

Semiquantitative analysis of the T_1w_ and T_2w_ images was performed by a radiology resident under supervision of a senior radiologist (C.S.M. with 3 years and R.H. with 10 years of experience in musculoskeletal imaging) visually similar as described previously [20, 24] using a four‐point visual scale for assessment of fat replacement/edema (T_1w/_T_2w_): Grade 1 homogeneous intermediate intensity (normal muscle), Grade 2 slight hypointensity/hyperintensity with patchy intramuscular signal intensity (SI) changes (<50% of muscle cross‐section), Grade 3 marked hypointensity/hyperintensity with patchy but widespread intramuscular SI changes (>50% of muscle cross‐section), and Grade 4 homogeneous hypointensity/hyperintensity in whole muscle.

Water T_2_ and FF values were extracted from the MESE ^1^H MRI data using an extended phase‐graph approach to compensate for stimulated echoes due to an inhomogeneous excitation field [25]. Muscles exhibiting a median FF > 60% were excluded from the evaluation of water T_2_ due to increasing fit instability.

Segmentation of seven individual lower leg muscle regions (gastrocnemius medialis/lateralis [GM/GL], soleus [SOL], tibialis anterior/posterior [TA/TP], extensor digitorum longus [EDL], and peroneus [PER]) as well as subcutaneous adipose tissue was performed based on the T_1w_^1^H MR images using DAFNE, a semi‐automatic image segmentation tool [26]. Segmentation masks were co‐registered/transformed to the quantitative image data (T_2_ mapping, FF, ^23^Na/^39^K MRI) using a nonrigid image co‐registration approach [27].

^23^Na and ^39^K raw datasets were reconstructed offline using a custom‐written Matlab tool (TheMathworks, Natick, USA) applying a nonuniform fast Fourier transform, and interpolated to a voxel size of 1 × 1 × 1 mm^3^. A Hamming filter was applied to reduce Gibb's ringing artifacts. Before determination of the aTSC and aTPC values, a region‐based partial volume correction (PVC) was performed using the individual segmentation masks as described before [9]. This PVC approach yields averaged ^23^Na/^39^K signal intensities for all segmented tissue regions, that is, individual muscles, subcutaneous adipose tissue, and the reference compartments. After PVC, a correction of relaxation biases was performed using the ^23^Na and ^39^K relaxation properties for muscle tissue and the reference solutions. Signal calibration was performed based on the five external reference compartments with known ion concentrations using a linear regression.

Finally, the determined aTSC and aTPC values for each muscle were corrected for fat replacement using the measured muscle‐averaged FF values and the expected ^23^Na and ^39^K concentrations in fatty tissue (aTSC_fat_ = 7.9 mM^4^ and aTPC_fat_ = 0 mM, respectively) according to

$$\begin{array}{c} \mathrm{aTS}C_{\mathrm{fc}}=\frac{\text{aTSC}-7.9\;\mathrm{mM}\cdot \mathrm{FF}}{\left(1-\mathrm{FF}\right)},\\ \;\mathrm{aTP}C_{\mathrm{fc}}=\frac{\text{aTPC}-0\;\mathrm{mM}\cdot \mathrm{FF}}{\left(1-\mathrm{FF}\right)}=\frac{\text{aTPC}}{\left(1-\mathrm{FF}\right)} \end{array}$$

As exact potassium concentrations in fat were not known a priori, negligible potassium concentrations were assumed. As for water T_2_, muscles with an FF > 60% were excluded from the evaluation of aTSC_fc_ and aTPC_fc_ due to the increasing fit uncertainty of the MESE fit for high FF values, and correspondingly higher uncertainty of the absolute FF values that would directly translate to higher uncertainty of aTSC_fc_ and aTPC_fc_.

### 2.5 Statistical Analysis

Due to the non‐normally distribution of the data, nonparametric tests were applied to analyze the data, which is expressed as median (interquartile range). Comparison of the aTSC and aTPC value between patients and healthy subjects was performed using a Wilcoxon rank‐sum test. Furthermore, muscles of FSHD patients were grouped according to their FF as weakly fatty replaced (FF < 10%), moderately fatty replaced (10% ≤ FF < 30%), and strongly fatty replaced (30% ≤ FF < 60%). Differences between these groups were assessed using the Kruskal–Wallis test for multiple comparison. Correlation between the quantitative MRI measures was examined using a Spearman's rank correlation test. A *p*‐value < 0.05 was considered significant.

## 3 Results

The determined scores for the muscle strength as well as clinical data of the patients can be found in Table 1. Corresponding semiquantitative scores for assessment of fatty replacement/edema are summarized in Table 3. Overall, fatty replacement of muscle tissue was scored highest in GM (median score = 3.5), while TP muscles of all but one patient were scored as normal (median score = 1).

**TABLE 3:** Semiquantitative scoring of fat replacement/edema based on T_1w_ and T_2w_^1^H MRI data in muscles of FSHD patients.

Patient	GM	GL	SOL	TA	TP	PER	EDL
1	4/1	3/3	3/1	4/1	1/1	1/1	4/1
2	4/1	2/3	3/1	4/1	1/1	1/2	4/1
3	1/1	1/1	1/1	1/1	1/1	1/1	1/1
4	1/1	1/1	1/1	1/1	1/1	1/1	1/1
5	3/2	1/2	1/1	3/3	1/1	1/1	3/3
6	3/3	1/2	1/1	1/1	1/1	1/1	1/1
7	4/1	4/1	3/2	4/1	2/1	3/2	4/1
8	1/1	1/1	1/1	1/1	1/1	1/1	1/1
9	1/1	1/1	1/1	1/1	1/1	1/1	1/1
10	4/1	1/3	3/4	2/2	1/1	1/1	1/1
11	1/1	1/1	1/1	1/1	1/1	1/1	1/1
12	3/3	3/3	4/1	1/2	1/1	1/2	1/1
13	1/1	1/1	1/2	1/1	1/1	1/1	1/1
14	2/1	1/1	1/2	3/3	1/1	1/1	3/2
Median	3.5/1	1/1	1/1	1/1	1/1	1/1	1/1

**FIGURE 1:**
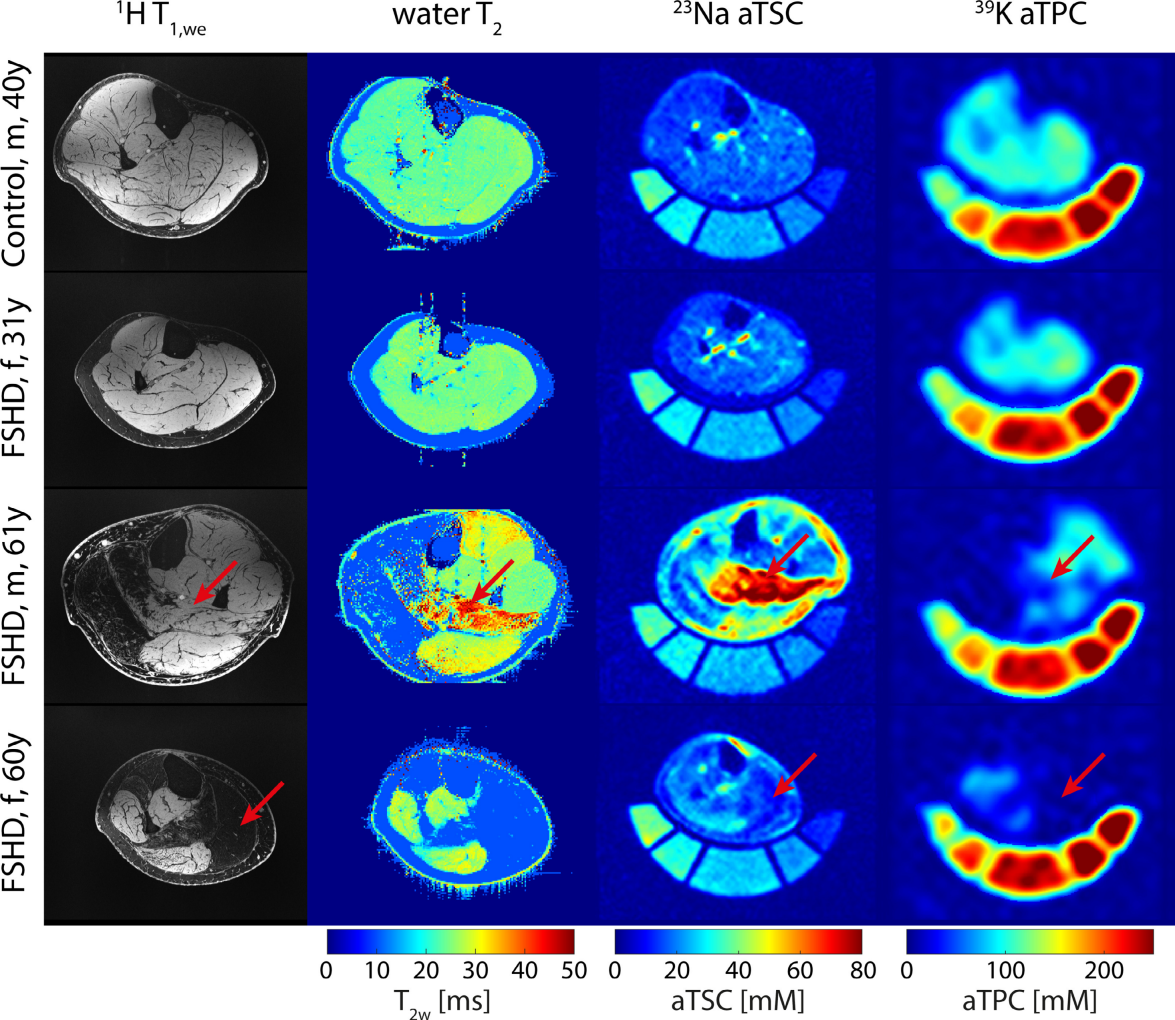
T_1w_^1^H MR images acquired with WE together with quantitative maps for proton water T_2_, ^23^Na apparent tissue sodium concentration (aTSC), and ^39^K apparent tissue potassium concentration (aTPC) (after correction for relaxation biases but before PVC) for three FSHD patients with varying disease progression (early disease stage with intramuscular FF < 10%, moderate fat replacement and strong fat replacement), and one healthy control. Non‐fat replaced muscles of FSHD patients showed similar aTSC and aTPC as well as water T_2_ relaxation times as healthy muscle tissue. In contrast, aTPC was reduced in muscles with beginning to moderate fat replacement, which also showed (highly) increased aTSC/water T_2_ (see arrows). Strongly fat replaced muscles showed negligible aTPC.

Figure 1 compares the axial view of T_1w_^1^H MRI images of three FSHD patients exhibiting a varying disease severity with the corresponding ^23^Na and ^39^K MRI images. Due to the very high spatial resolution, fine structures and patterns of fatty replacement were well visible in the T_1w_‐weighted images. In addition, ^39^K MRI was highly sensitive to fat replacement and depicted the muscle degeneration. Particularly, even in regions with normally appearing or increased ^23^Na MR signal, a strongly reduced ^39^K MRI signal could be observed.

**FIGURE 2:**
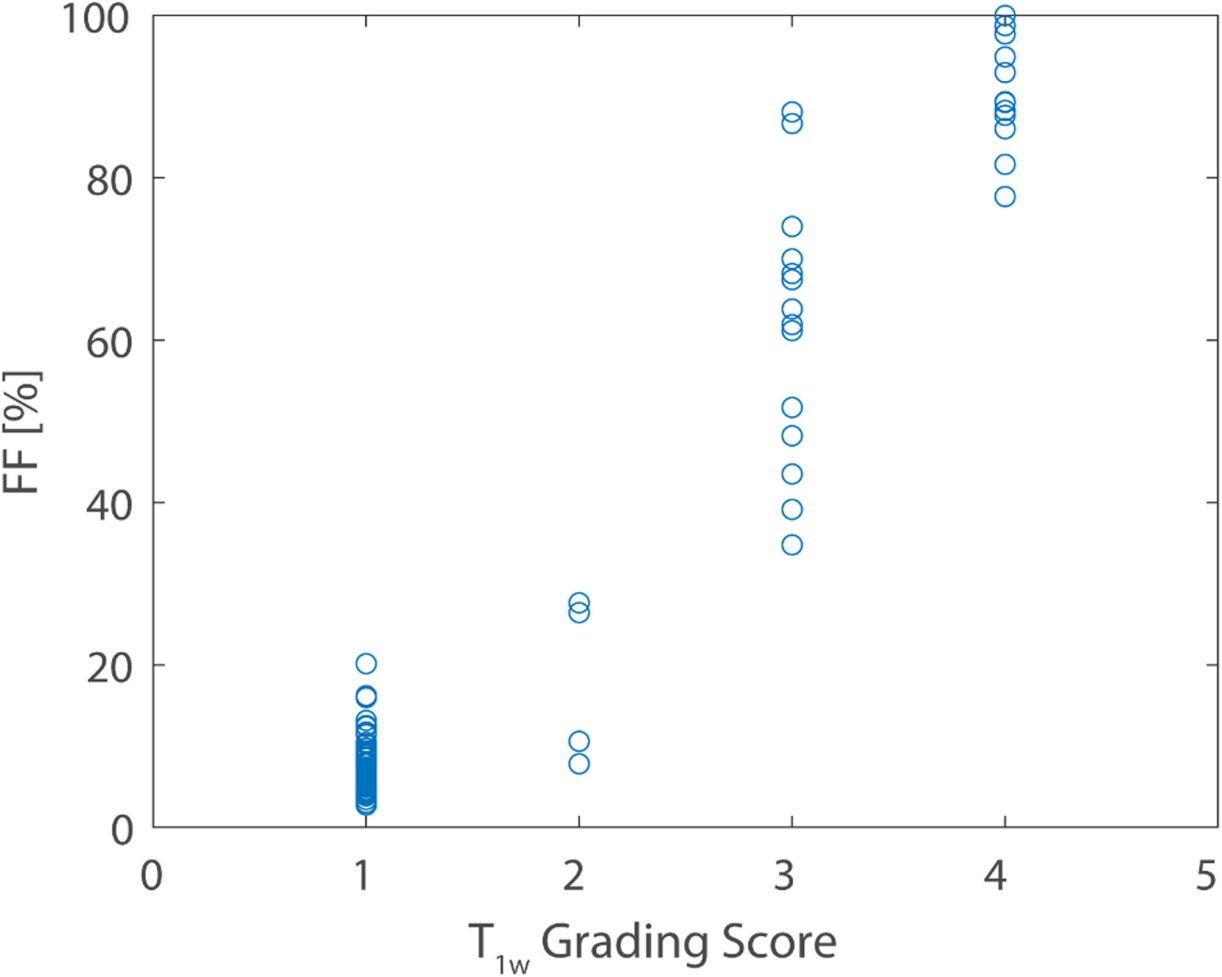
Comparison of semiquantitative grading scores for fat replacement based on T_1w_ images (compare Table 3) and FF values extracted from MESE T_2_ fit. Overall, FF values and grading scores were consistent (Spearman rho = 0.79, *p* < 0.001), especially in the very low and very high FF range. Muscles with a T_1w_ grading score of 3 showed a higher variation of measured FF values. There was one muscle in which a very strong edema was partly misinterpreted as increased FF by the T_2_ fit (see Figure S1).

Overall, the semiquantitative scores of the fat replacement based on the T_1w_ images and the FF values extracted from the MESE T_2_ fit were consistent (see Figure 2). However, there was one muscle in which a very strong edema was partly misinterpreted as increased FF by the EPG fit (SOL muscle of patient 10, see Figure S1).

**FIGURE 3:**
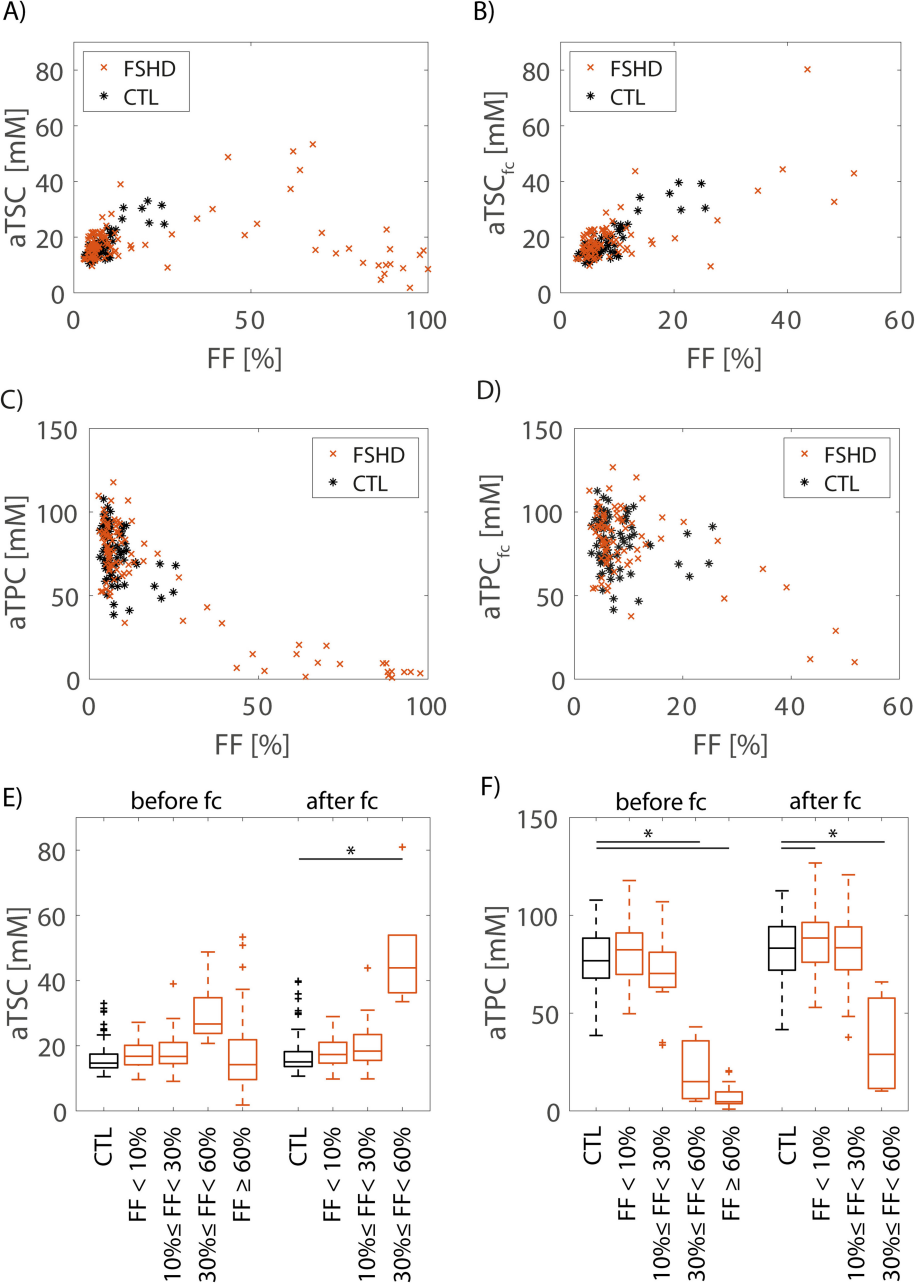
Dependence of measured aTSC (A, B) and aTPC (C, D) values before and after fat correction on FF values measured by EPG ^**1**^H T_2_ fit. Each data point represents an individual muscle (seven muscles evaluated per patient/control). Before fat correction, aTPC values strongly decreased with increasing FF and were close to zero for almost fully fatty‐replaced muscle (Spearman rho = −0.51, *p* < 0.001). After fat correction, aTSC_fc_ values increased and aTPC_fc_ values decreased with increasing FF (aTSC_fc_: Spearman rho = 0.55, *p* < 0.001; aTPC_fc_: Spearman rho = −0.18, *p* = 0.02). Only muscles with FF < 60% were included in the evaluation after fat correction due to the potential uncertainty in FF values extracted from the T_2_ fit in regions of very high fat replacement. When grouped depending on the FF range, muscles with a FF between 30% and 60% showed significantly increased aTSC and reduced aTPC compared with healthy controls (CTL), both before and after fat correction (fc) (E, F). Significant differences compared with healthy controls are marked with an asterisk.

Figure 3 shows the dependence of the measured aTSC and aTPC values of individual muscles after PVC on the FF derived from the EPG fit. While aTSC values were increased for medium FFs and decreased for very high FFs, aTPC values strongly decreased with FF already at medium FF values (see Figure 3A,C). For very high FFs (> 80%), the median aTPC was close to zero (4.3 mM [2.7 mM]), and the median aTSC was 10.0 mM (5.9 mM). To calculate aTSC and aTPC values for remaining muscle tissue, a correction for reduced sodium and potassium concentrations in fatty tissue was performed (see Figure 3B,D). In healthy controls, the median fat‐corrected correction concentrations were aTSC_fc_ = 15.0 mM (4.5 mM), and aTPC_fc_ = 83.2 mM (22.3 mM). When grouping muscles of patients depending on the FF range, muscles with an FF between 30% and 60% showed highest aTSC_fc_ (42.3 mM [17.6 mM]) and reduced aTPC_fc_ (28.9 mM [46.2 mM]) after fat correction (see Figure 3E,F). aTPC_fc_ values in muscles of FSHD patients with low or moderate fat replacement (< 30%) were in a similar range as corresponding values in healthy muscle tissue (FF < 10%: aTPC_fc_ = 88.4 mM [20.3 mM]; 10% ≤ FF < 30%: aTPC_fc_ = 83.4 mM [21.9 mM]).

**FIGURE 4:**
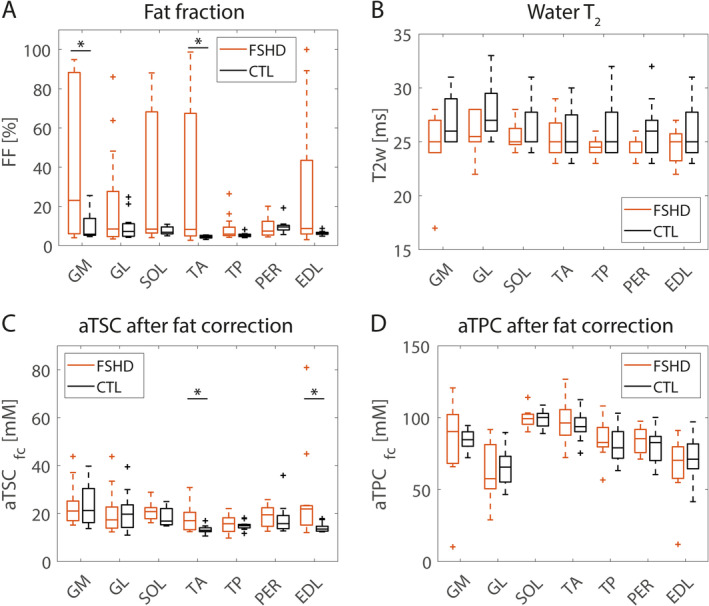
Comparison of FF (A), water T_2_ (B), aTSC (C), and aTPC (D) values after fat correction in individual muscles of FSHD patients and healthy controls. For the evaluation of water T_2_ and fat corrected aTSC and aTCP values, only muscles with FF below 60% were considered. Significant differences between FSHD patients and healthy controls are marked with *.

**FIGURE 5:**
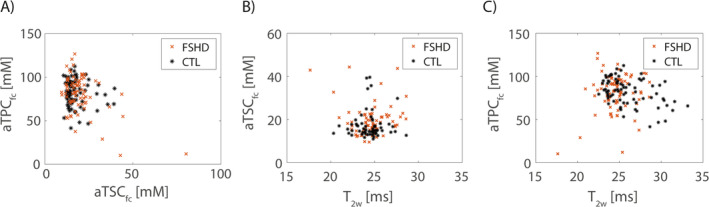
Relation between the quantitative MR measures derived from ^**23**^Na/^**39**^K and ^**1**^H MRI. There were no significant correlations between fat‐corrected aTSC and aTPC (A), fat‐corrected aTSC and water T_2_ (B), and fat‐corrected aTPC and water T_2_ (C).

A comparison between the quantitative values measured in individual lower leg muscles of FSHD patients and healthy controls is shown in Figure 4. For the evaluation of water T_2_, aTSC_fc_, and aTCP_fc_, only muscles with FF < 60% were considered. Corresponding median FF, water T_2_, aTSC_fc_, and aTPC_fc_ values are listed in Table 4. GM of patients showed highest fat replacement with a median FF of 23.1% (82.3%). Overall, FF of patients was significantly increased in GM and TA muscles compared with healthy controls (*p* = 0.046/0.008 for GM/TA). In contrast, water T_2_ values in FSHD patients were comparable with controls for all muscle regions. aTSC_fc_ was significantly increased in TA and EDL muscles of FSHD patients compared with healthy controls (*p* = 0.033/0.010). There were no significant differences in aTPC_fc_ values of individual muscles between patients and healthy controls. Figure 5 visualizes the relation between the individual quantitative MRI measures. There were no significant correlations between aTSC, aTPC, and water T_2_.

**TABLE 4:** Median (interquartile range) for FF, water T_2_, aTSC_fc_, and aTPC_fc_ values in FSHD patients and healthy subjects for all seven examined muscle regions. For evaluation of water T_2_, aTSC_fc_, and aTPC_fc_, only muscles with FF < 60% were considered. Values with significant differences between FSHD patients and healthy controls are marked with (*).

Muscle	FF (%)		Water T_2_ (ms)		aTSC_fc_ (mM)		aTPC_fc_ (mM)	
	FSHD	CTL	FSHD	CTL	FSHD	CTL	FSHD	CTL
GM	23.1^(*)^ (82.3)	5.8 (8.6)	25 (3)	26 (4)	34.0 (8.1)	21.2 (14.0)	90.3 (82.6)	84.7 (10.1)
GL	8.5 (23.0)	7.2 (6.4)	25.5 (3)	27 (3.5)	30.7 (8.6)	19.7 (9.4)	57.6 (36.9)	65.7 (17.9)
SOL	8.4 (61.8)	6.7 (3.5)	25 (1.5)	25 (2.75)	7.0 (4.6)	16.8 (6.7)	99.4 (7.0)	100.1 (8.7)
TA	8.3^(*)^ (62.5)	4.6 (1.4)	25 (2.75)	25 (3.5)	17.7^(*)^ (7.3)	13.0 (1.7)	96.3 (17.7)	93.8 (9.7)
TP	5.9 (4.3)	5.2 (1.2)	24.5 (1)	25 (3.75)	13.5 (5.7)	15.0 (1.4)	82.8 (13.5)	79.1 (18.5)
PER	7.5 (7.0)	9.6 (2.4)	25 (1)	26 (3)	16.1 (7.7)	15.7 (5.5)	85.5 (16.1)	82.8 (16.8)
EDL	8.8 (37.5)	6.4 (1.0)	25 (2.5)	25 (3.75)	22.0^(*)^ (8.1)	13.5 (2.0)	70.4 (22.0)	71.2 (17.2)

## 4 Discussion

This work investigated the feasibility of aTPC determination in partly fat‐replaced dystrophic skeletal muscle tissue using ^39^KMRI at 7 T. We observed a strong dependence of the ^39^K MRI signal on the disease state, with strongly reduced aTPC values measured in fat replaced muscles. A correction for reduced potassium concentration in fat tissue allowed assessment of aTPC_fc_ in remaining muscle tissue of FHSD patients, revealing reduced aTPC_fc_ values in muscles with advanced disease state (corresponding to FF ≥ 30%). Overall, the reduction in muscular potassium concentration was greater than the expected reduction due to fat replacement, indicating that reduced aTPC_fc_ might be associated with the pathology of muscular dystrophies.

Overall, aTPC showed a stronger dependence on fat replacement than aTSC. Typically, the potassium concentration in fat tissue is assumed to be negligible as 98% of the body potassium are located within the intracellular space [28]. In this work, the aTPC values measured in almost fully fatty replaced muscles (FF > 80%) were close to the potassium concentrations typically found within extracellular fluids (plasma [K^+^] normally in the range of 3–5 mM [29]).

Other MRI studies mostly evaluated the FF or T_2_‐weighted STIR images in FSHD patients [30, 31, 32, 33]. Non‐proton MRI and water relaxometry, as performed in this work, might additionally reveal information on the underlying pathophysiological changes. An enhanced aTSC has already been suggested as an indicator of disease activity in different types of muscular dystrophies [3, 4, 5]. The results of our study are in accordance with the findings of these studies as we also observed a strongly increased aTSC_fc_ in muscles with medium to high FF. Additionally, we evaluated the aTPC_fc_, which has not been investigated in patients with muscular dystrophies so far. We could demonstrate that besides elevated ^23^Na signals [5], muscle tissue of FSHD patients shows decreased aTPC_fc_ values in advanced disease stages. This might be caused by decreased intracellular potassium concentration, an increased extracellular volume fraction while maintaining constant intracellular/extracellular ion concentrations, or a combination of both in the affected muscle tissue. Further studies applying ^39^K MRI in combination with other (MRI) measures to dystrophic muscle tissue will be required to fully explain the origin of the observed changes in aTPC_fc._

To assess the aTPC in the remaining muscle tissue, a fat correction was performed analogously to the correction of aTSC as reported before [4, 5]. For this correction, we used FF values resulting from the EPG water T_2_ fit. At 3 T, FF maps extracted from T_2_ mapping data have been shown to be in good agreement with three‐point Dixon measurements [25, 34]. However, FF values resulting from EPG water T_2_ fit might be biased, especially in regions of strong edema and high fat replacement. This is why we excluded muscles with a median FF > 60% in our evaluation of fat‐corrected aTSC and aTPC as well as water T_2_. Still, we might have incorrectly included muscles with FF > 60% in the evaluation due to potential uncertainties of the absolute FF values, which would directly translate to the aTSC_fc_ and aTPC_fc_ values. In general, a chemical‐shift‐based approach for fat‐water separation might result in a more precise FF determination, corresponding to an enhanced quantification accuracy for aTSC_fc_ and aTPC_fc_. At the time the study was conducted, no optimized chemical shift‐based fat‐water separation was available at our 7 T system so that values extracted from the MESE T_2_ fit were the only available estimate for the muscular FF. As quantitative ^1^H MRI approaches are generally more challenging at ultra‐high field (e.g. due to inhomogeneities in *B*_0_/*B*_1_) [35], it might be advantageous to combine ^39^K/^23^Na MRI at 7 T with more stable and reliable ^1^H MRI sequences for skeletal muscles at 3 T.

Another benefit of using a more reliable FF determination approach would be that muscles with very high FF could also be included in the aTSC_fc_ and aTPC_fc_ evaluation. For example, Gerhalter et al. performed fat correction of the ^23^Na MR signal in muscles of FSHD patients with strong fat replacement using FF values measured with a Dixon‐based approach at 3 T [5]. Still, the fat correction of the aTPC might fail in muscles with FF values close to 100%. In these muscles, the uncorrected ^39^K MR signal is close to zero and small uncertainties in the uncorrected ^39^K MR signal or FF values might translate into large uncertainties in the resulting aTPC_fc_ values. In contrast to previous studies performed in healthy skeletal muscle tissue, the aTPC values differed between individual lower leg muscle regions both in FSHD patients and healthy controls. Especially, reduced aTPC values were measured in GL muscles compared with GM, SOL, TA, and TP. This might be caused by imperfect image co‐registration between the ^1^H and ^39^K/^23^Na MRI data leading to uncertainties in aTPC determination. The difference in deformation of the calf caused by the two RF coil setups used for ^1^H and ^39^K/^23^Na MRI was strongest in the GL muscle region, which was lying directly on top of the RF coils. While uncertainties due to imperfect co‐registration were strongest in GL, the overall results for aTSC/aTPC did not change by removing this muscle from the evaluation (data not shown). Better co‐registration between ^1^H and ^39^K/^23^Na MRI data might also enable a voxel‐wise correction of the FF instead of correction on a muscle basis as performed in this work. Still, a voxel‐wise correction of the ^39^K/^23^Na signal intensities would not be compatible with the region‐based partial volume correction approach applied in this work.

Another limitation of applying a region‐based PVC for aTSC and aTPC determination is that heterogeneity fat replacement within individual muscles—as often observed in FSHD patients—cannot be reflected. Several muscles of FSHD patients had a median FF < 60% but still showed patches of very high FF (see Figure S2). A possible solution to overcome this limitation would be to further divide the segmentation masks, for example, into regions of higher and lower fat replacement. This would however require a subjective assessment of the borders of such regions and might therefore reduce the reproducibility of the evaluation approach.

The healthy controls examined in this work were age‐matched to the FSHD patients. Thus, some healthy controls had naturally slightly increased muscular FF according to their age (> 60 years). It would be highly interesting to investigate muscle tissue of even older; otherwise, healthy subjects to separate changes in measured aTPC due to naturally increased FF from pathological changes in FSHD. However, the probability for medical conditions known to alter the tissue ion concentrations (e.g., hypertension) as well as the application of exclusion criteria for 7 T MRI (e.g., metallic implants) increase with age, so that recruiting of suitable subjects for such investigations is challenging.

Finally, ^23^Na and ^39^K relaxation times might be altered in dystrophic muscle, especially in regions with edema, which might influence the relaxation correction and PVC of the ^23^Na and ^39^K MRI data. An individual measurement of ^23^Na and ^39^K relaxation times might therefore improve aTSC and aTPC determination; however, it is very time‐consuming (at least 30 min) and was therefore not performed in this study.

## 5 Conclusion

^39^K MRI can provide interesting additional insights into the patho‐physiological processes in muscle dystrophy such as FSHD. As the potassium concentration is usually tightly regulated, alterations in ^39^K MRI might therefore serve as an additional marker for muscular dystrophies. However, quantitative analysis of aTPC in dystrophic muscle tissue requires correction of partial volume effects and FF to reliably assess potassium content in remaining muscle tissue.

## Supporting information


**Figure S1** Comparison of *T*_1w_/*T*_2w_ images and fat fraction (FF) map extracted from EPG T_2_ fit of patient 10. The strong edema in the soleus muscle (semiquantitative score = 4 based on *T*_2w_ image) was partly misinterpreted as increased FF by the EPG fit. As the resulting median FF was > 60%, this muscle was excluded from the evaluation of aTSC_fc_/aTPC_fc_ and water *T*_2_.
**Figure S2**: Distribution of measured fat fraction (FF) values in muscles with median FF < 60% of three exemplary FSHD patients. The marked muscle regions had a median FF < 60%, however with a significant proportion of voxels with an FF > 60% (evaluated as the cumulative probability of FF > 60%). This heterogeneity in fat replacement is not reflected by the used aTSC and aTPC quantification approach applying on a region‐based partial volume correction, which assumes constant signal distributions over the entire muscle regions.

## Data Availability

Research data are not shared.
