# Supplementary material for: Determination of Tissue Potassium and Sodium Concentrations in Dystrophic Skeletal Muscle Tissue Using Combined Potassium (39K) and Sodium (23Na) MRI at 7 T
Source: NMR Biomed. 2025 Feb 17;38(4):e70009. doi: 10.1002/nbm.70009 (PMC11833145; doi:10.1002/nbm.70009)
Supplement: Supplementary file 1 — Figure S1 Comparison of T 1w/T 2w images and fat fraction (FF) map extracted from EPG T2 fit of patient 10. The strong edema in the soleus muscle (semiquantitative score = 4 based on T 2w image) was partly misinterpreted as increased FF by the EPG fit. As the resulting median FF was > 60%, this muscle was excluded from the evaluation of aTSCfc/aTPCfc and water T 2. Figure S2: Distribution of measured fat fraction (FF) values in muscles with median FF < 60% of three exemplary FSHD patients. The marked muscle regions had a median FF < 60%, however with a significant proportion of voxels with an FF > 60% (evaluated as the cumulative probability of FF > 60%). This heterogeneity in fat replacement is not reflected by the used aTSC and aTPC quantification approach applying on a region‐based partial volume correction, which assumes constant signal distributions over the entire muscle regions. [file NBM-38-e70009-s001.docx]

**Supplementary Information**

*
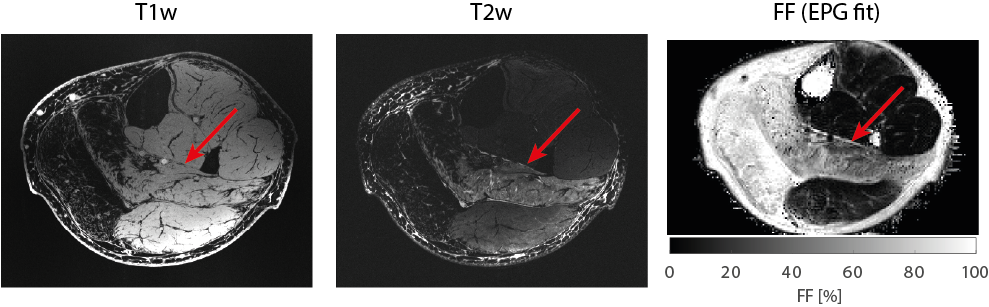
*

**Supplementary Information Figure S1**: Comparison of T_1w_/T_2w_ images and fat fraction (FF) map extracted from EPG T_2_ fit of patient 10. The strong edema in the soleus muscle (semi-quantitative score = 4 based on T_2w_ image) was partly misinterpreted as increased FF by the EPG fit. As the resulting median FF was > 60%, this muscle was excluded from the evaluation of aTSC_fc_/aTPC_fc_ and water T_2_.


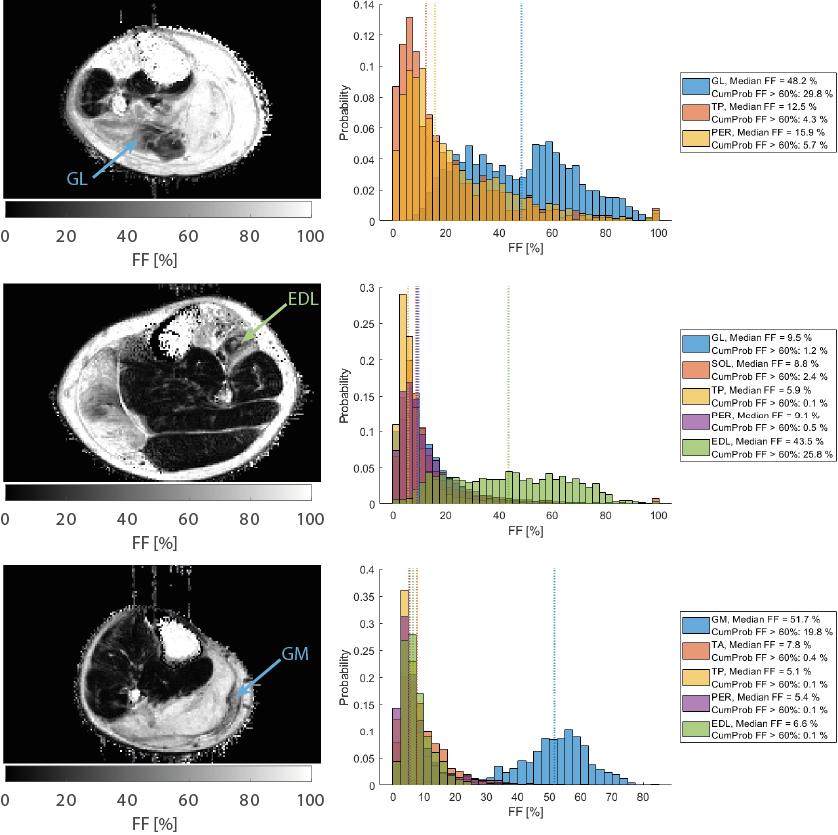


**Supplementary Information Figure S2**: Distribution of measured fat fraction (FF) values in muscles with median FF < 60% of three exemplary FSHD patients. The marked muscle regions had a median FF < 60%, however with a significant proportion of voxels with an FF > 60% (evaluated as the cumulative probability of FF > 60%). This heterogeneity in fat replacement is not reflected by the used aTSC and aTPC quantification approach applying on a region-based partial volume correction, which assumes constant signal distributions over the entire muscle regions.
